# Exploring the gateway hypothesis of e-cigarettes and tobacco: a prospective replication study among adolescents in the Netherlands and Flanders

**DOI:** 10.1136/tobaccocontrol-2021-056528

**Published:** 2021-07-05

**Authors:** Thomas Martinelli, Math J J M Candel, Hein de Vries, Reinskje Talhout, Vera Knapen, Constant P van Schayck, Gera E Nagelhout

**Affiliations:** 1 IVO, The Hague, The Netherlands; 2 Tranzo, Tilburg School of Social and Behavioral Sciences, Tilburg University, Tilburg, The Netherlands; 3 Department of Methodology and Statistics, Maastricht University, Maastricht, The Netherlands; 4 Department of Health Promotion, Maastricht University, Maastricht, The Netherlands; 5 Laboratory for Health Protection Research, National Institute for Public Health and the Environment (RIVM), Bilthoven, The Netherlands; 6 Department of Family Medicine, Maastricht University, Maastricht, The Netherlands

**Keywords:** electronic nicotine delivery devices, public policy, harm reduction

## Abstract

**Background:**

Studies demonstrated that adolescent e-cigarette use is associated with subsequent tobacco smoking, commonly referred to as the *gateway effect*. However, most studies only investigated gateways from e-cigarettes to tobacco smoking. This study replicates a cornerstone study revealing a positive association between both adolescent e-cigarette use and subsequent tobacco use; and tobacco and subsequent e-cigarette use in the Netherlands and Flanders.

**Design:**

The longitudinal design included baseline (n=2839) and 6-month (n=1276) and 12-month (n=1025) follow-up surveys among a school-based cohort (mean age: 13.62). Ten high schools were recruited as a convenience sample. The analyses involved (1) associations of baseline e-cigarette use and subsequent tobacco smoking among never smokers; (2) associations of e-cigarette use frequency at baseline and tobacco smoking frequency at follow-up; and (3) the association of baseline tobacco smoking and subsequent e-cigarette use among non-users of e-cigarettes.

**Findings:**

Consistent with prior findings, baseline e-cigarette use was associated with higher odds of tobacco smoking at 6-month (OR=1.89; 95% CI 1.05 to 3.37) and 12-month (OR=5.63; 95% CI 3.04 to 10.42) follow-ups. More frequent use of e-cigarettes at baseline was associated with more frequent smoking at follow-ups. Baseline tobacco smoking was associated with subsequent e-cigarette use (OR=3.10; 95% CI 1.58 to 6.06 at both follow-ups).

**Conclusion:**

Our study replicated the positive relation between e-cigarette use and tobacco smoking in both directions for adolescents. This may mean that the gateway works in two directions, that e-cigarette and tobacco use share common risk factors, or that both mechanisms apply.

## Introduction

Debates about electronic cigarettes are dividing many of those concerned with tobacco control. Proponents of e-cigarettes point at evidence which suggests that e-cigarettes are an effective smoking cessation aid^1^ and that e-cigarette use is substantially less harmful than tobacco smoking.^2^ Therefore, e-cigarettes could dramatically reduce disease and death caused by smoking.^3 4^ However, opponents are concerned that e-cigarettes attract new generations of youth into nicotine addiction,^5^ that most e-cigarette users simultaneously use tobacco^6 7^ and that it acts as a gateway to smoking tobacco.^8 9^

The *gateway* or *stepping stones* theory originates from the 1970s as a mix of academic and popular explanations of the observed sequence from cannabis use to other illicit drug use.^10–12^ While initially descriptive, the theory was also used to explain causal relationships of substance use.^13^ However, the theory was and remains controversial as it fails to exclude alternative explanations, particularly the notion that all substance use is associated with shared characteristics of individuals, especially a propensity to use drugs.^14^ More recently, the gateway theory is applied to e-cigarettes in both policy and research.^15^ The European Tobacco Products Directive, for example, states that ‘Electronic cigarettes can develop into a gateway to nicotine addiction and ultimately traditional tobacco consumption’.^16^

Various cohort studies show that in the last decade e-cigarette use among adolescents increased while tobacco use decreased.^17–20^ Simultaneously, longitudinal studies show that e-cigarette use is associated with initiation of tobacco smoking. The first study that revealed such association among adolescents was published in 2015.^21 22^ These findings generated ample media attention and led to restrictions for e-cigarettes in the USA, prohibiting the sale to persons under 18 years.^23^ Since then, cohort studies^3 17 20 24–28^ have followed finding similar associations, including in the Netherlands.^29^ Despite the above research into the relation between e-cigarette use and tobacco smoking, only few studies examined the reverse relation between tobacco smoking and initiation of e-cigarette use,^21 30 31^ which has also been found for tobacco and alcohol use, for example.^32^

**Figure 1:**
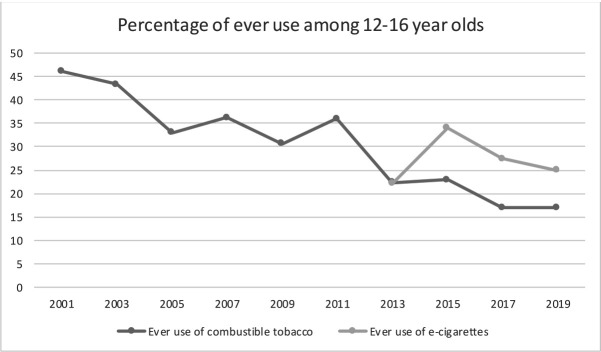
Ever use of tobacco and e-cigarettes in Dutch youth. Source: Health Behaviour in School-aged Children (HBSC 2001, 2005, 2009, 2013, 2017) and Peilstationsonderzoek (Peil 2003, 2007, 2011, 2015, 2019).

In this paper, we present a replication study with new data using the same protocol as the cornerstone study by Leventhal *et al*.^21 22^ Replication is crucial to the scientific method as it enables one to build on demonstrated and confirmed findings.^33^ We collected data among adolescents in the Netherlands and Flanders, the Dutch-speaking region of Belgium. Both regions have similar legislation in which e-cigarettes (with and without nicotine) are treated as tobacco products, meaning that sales to minors (under 18 years) and advertising are prohibited.^34 35^ This is different in the original study setting in the USA, where few federal restrictions on e-cigarette marketing existed at the time of the study and some leading e-cigarette brands have been investigated for particularly targeting youth.^36–39^ In the Netherlands, in 2019, 25% of youth between 12 and 16 years old had ever tried an e-cigarette and 17% had ever tried smoking tobacco^19^ (see figure 1). Currently, more youth experiment with e-cigarettes than with tobacco. With this paper, we aim to answer the following research questions:

1. Is e-cigarette use associated with subsequent combustible tobacco use?
2. Is e-cigarette use frequency associated with subsequent tobacco smoking frequency?
3. Is combustible tobacco use associated with subsequent e-cigarette use?

## Methods

### Participants and procedure

Data were collected with online surveys between September 2018 and December 2019 throughout the Netherlands and Flanders. Since high schools are very hard to recruit for research, due to the large number of study requests and ongoing studies, we used multiple recruitment strategies. The research was presented as a study on smoking, alcohol, drugs and other risk behaviours. Different national and regional organisations (including addiction services, school health promotion and youth organisations) were approached to help contact schools. A representative selection of 82 schools were approached by telephone, of which 50 received recruitment packages. At 14 schools we reached the right contacts by phone. Another 10 schools were approached through informal networks of the research group. Lastly, a mass recruitment e-mail with reminder was sent to 580 schools in the Netherlands and 1343 in Flanders (Belgium). In total, 10 schools responded with interest in our study, and of those, all agreed to participate, including eight schools in the Netherlands and two in Flanders. The schools were provided with informed consent forms about the study, which they disseminated among parents and students. Students enrolled through passive consent and were excluded if they or their parents refused participation. Data collection involved three waves that took place 6 months apart. We provided participating schools with links to online surveys at each wave and the schools administered the surveys in the classes. Since recruitment of schools was difficult, some schools enrolled later. This meant that some schools only participated 6 months in the study and did not complete all three surveys. This partly explains the attrition rates across follow-ups (see online supplemental figure 1). Other reasons for attrition were that some students left the school (either to change schools or because they graduated), some schools had shop classes (such as wood or metal shop class, which meant they were at other locations), illness at the day of the survey and, lastly, one school could not be reached to conduct a follow-up despite many reminders.

10.1136/tobaccocontrol-2021-056528.supp1 Supplementary data

### Measures

All measures are the same as in the replicated study^21^ and possess adequate psychometric properties in youth samples.^40–44^

#### E-cigarette and combustible tobacco use

Youth Risk Behavior Surveillance (YRBS)^40^ and Monitoring the Future Surveys (MTF)^41^ based items measured lifetime (ever) and past 6-month use (yes/no) of e-cigarettes and combustible tobacco products, which included: combustible cigarettes (including ‘even a few puffs’), full-size cigars, little cigars/cigarillos and hookah waterpipes. Lifetime (ever vs never) e-cigarette use at baseline was the primary exposure variable. Outcome variables were any use in the last 6 months (yes/no) of: (1) any combustible tobacco product; (2) combustible cigarettes; (3) cigars (full-size cigars or little cigars); (4) hookah; and (5) the number of different combustible tobacco products (range: 0–3). The terms ‘ever-smokers’ and ‘never-smokers’ refer to participants who either have ever or never used any of the three combustible tobacco products, respectively. The four-level continuous e-cigarette use frequency variable was categorised as never, prior (ever use with no past 30-day use), infrequent (1–2 days during the past 30 days) or frequent (≥3 days during the past 30 days). The cigarette use frequency variable consisted of non-smokers (smoked 0 day in the past 30 days); infrequent smokers (smoked 1–2 days in the past 30 days); and frequent smokers (smoked ≥3 days in the past 30 days).

#### Covariates

Variables that are potentially associated with risk of combustible tobacco use initiation based on previous literature^45–50^ were selected a priori as covariates, as they potentially overlap with both e-cigarette use and tobacco use. Covariate categories are described below.

#### Sociodemographics

Age, gender, ethnicity and highest parental education represented sociodemographics (table 1 contains response categories). In the main analysis, Belgian participants from Belgian (Flanders) schools were coded as Dutch students, as they belonged to the ethnic majority (native) group of their school.

**Table 1:** Sample characteristics by baseline ever e-cigarette use status among baseline never smokers

Outcome	Baseline e-cigarette use			Contrast by ever e-cigarette use P value
	Overall (n=2185)	Never use (n=1994)	Ever use (n=191)	
**Sociodemographics**				
Sex, n (%)				<0.001*
Girls	1160 (53.1)	1097 (55.0)	63 (33.0)	
Boys	1025 (46.9)	897 (45.0)	128 (67.0)	
Age, M (95% CI)	13.62 (13.56 to 13.67)	13.57 (13.51 to 13.62)	14.16 (13.99 to 14.34)	<0.001†
Ethnicity, n (%)				0.063*
Dutch	1978 (90.5)	1816 (91.1)	162 (84.8)	
Belgian	76 (3.5)	65 (3.3)	11 (5.8)	
Polish	8 (0.4)	7 (0.4)	1 (0.5)	
Turkish	30 (1.4)	24 (1.2)	6 (3.1)	
German	5 (0.2)	5 (0.3)	0	
British	4 (0.2)	4 (0.2)	0	
Moroccan	16 (0.7)	13 (0.7)	3 (1.6)	
Chinese	6 (0.3)	4 (0.2)	2 (1.0)	
Italian	5 (0.2)	5 (0.3)	0	
Spanish	5 (0.2)	5 (0.3)	0	
Other, don’t want to say or unknown	52 (2.4)	47 (0.2)	1 (0.5)	
Highest parental education, n (%)				0.029‡
Graduate degree	271 (12.4)	248 (12.4)	23 (12.0)	
College graduate	478 (21.9)	426 (21.4)	52 (27.2)	
Secondary higher education	368 (16.8)	347 (17.4)	21 (11.0)	
Vocational education	301 (13.8)	267 (13.4)	34 (17.8)	
Secondary vocational education	211 (9.7)	189 (9.5)	22 (11.5)	
Lower vocational education	42 (1.9)	34 (1.7)	8 (4.2)	
Primary school	14 (0.6)	13 (0.7)	1 (0.5)	
None	23 (1.1)	22 (1.1)	1 (0.5)	
Don’t know	477 (21.8)	448 (22.5)	29 (15.2)	
**Environmental factors**				
Lives with both biological parents, n (%)	1729 (79.1)	1606 (80.5)	123 (64.4)	<0.001*
Family history of smoking, n (%)	1522 (69.7)	1364 (68.4)	158 (82.7)	<0.001*
Peer smoking, M (95% CI)	1.32 (1.29 to 1.36)	1.27 (1.24 to 1.31)	1.84 (1.64 to 2.04)	<0.001†
**Intrapersonal factors**				
CESD-Depressive symptoms, M (95% CI)	9.91 (9.55 to 10.27)	9.69 (9.32 to 10.07)	12.19 (10.84 to 13.55)	<0.001†
TCI-Impulsivity, M (95% CI)	7.99 (7.87 to 8.10)	8.08 (7.97 to 8.20)	6.97 (6.55 to 7.39)	<0.001†
Ever substance use, n (%)	757 (34.6)	617 (30.9)	140 (73.3)	<0.001*
Delinquent behaviour, M (95% CI)	14.39 (14.23 to 14.55)	14.13 (13.97 to 14.29)	17.08 (16.32 to 17.85)	<0.001†
Smoking susceptibility, M (95% CI)	3.47 (3.43 to 3.51)	3.40 (3.36 to 3.44)	4.20 (4.00 to 4.41)	<0.001†
Smoking expectancies, M (95% CI)	6.90 (6.85 to 6.95)	6.93 (6.88 to 6.98)	6.53 (6.36 to 6.71)	<0.001†

*χ^2^ test.

†Independent samples t-test.

‡Spearman’s r test.

CESD, Center for Epidemiologic Studies Depression Scale; M, mean; TCI, Temperament and Character Inventory.

#### Environmental variables

Environment indicators included family living situation, assessed by asking: ‘Who do you live with most of the time?’ (both biological parents/other).^46^ Family history of smoking was assessed by asking: ‘Does anyone in your immediate family (brothers, sisters, parents, or grandparents) have a history of smoking cigarettes?’ (yes/no). Peer smoking was measured by asking: ‘In the last 30 days, how many of your five closest friends have smoked cigarettes?’ (range: 0–5).^51^

#### Intrapersonal factors

Personality traits, mental health and psychological processes that are linked to risky behaviour, experimentation and smoking were assessed. Depressive symptoms were assessed through the 20-item Center for Epidemiologic Studies Depression Scale^43^ composite sum of past week frequency ratings (eg, 0=Rarely or none of the time (0–1 day) to 3=Most or all of the time (5–7 days)). Impulsivity was assessed with Temperament and Character Inventory^52^ Impulsivity subscale sum score (eg, ‘I often do things based on how I feel at the moment’; range: 0–5). Lifetime use of other (non-nicotine or tobacco) substances was assessed with the MTF and YRBS items on ever use (yes/no) of alcohol and 13 other substances. Delinquent behaviour^53^ was assessed by calculating a mean of frequency ratings for engaging in 11 different behaviours (eg, stealing, lying to parents; 1=Never to 6=Ten or more times) in the past 6 months. Susceptibility to smoking was measured by using a mean of a three-item index^44^ (eg, ‘Would you try smoking a cigarette if one of your best friends offered it to you?’ (1=Definitely not, 4=Definitely yes)). Smoking outcome expectancies were assessed by a mean of the two responses for ‘I think I might enjoy (…) smoking’ and (reversed) ‘I think I might feel bad (…) from smoking’ (1=Strongly disagree, 4=Strongly agree).^54^

### Data analysis

Following the protocol of Leventhal *et al*,^21^ prevalence and associations of lifetime (ever vs never) e-cigarette use and lifetime (ever vs never) combustible tobacco use in the overall baseline sample were first analysed. Subsequently, study attrition and descriptive statistics in the sample of baseline never smokers were reported. For the primary analyses, separate generalised linear mixed models^55^ for each outcome were used with the follow-up data at two time points (6 and 12 months) as repeated measurements. For research question 1, each binary outcome (eg, any lifetime combustible tobacco product, cigarettes, cigars, hookah) was analysed with logistic mixed regression, whereas the ‘number of combustible products’ outcome was analysed with ordinal mixed regression. All models included baseline e-cigarette ever use, school and time (6-month vs 12-month follow-up) as fixed effects and were fit with and without adjustment for all covariates. To examine whether the association between baseline e-cigarette use and combustible tobacco use differed across follow-ups, the baseline ‘e-cigarette × time’ interaction term was added to each model. If this interaction was significant, the association between e-cigarette use and the outcome was examined separately for the 6 and 12-month follow-ups, including differences in outcome for ever and never use of e-cigarettes at baseline. If not significant, the interaction term was removed from the model, and associations between baseline e-cigarette use and the outcome were averaged across both follow-ups. Participants with missing data on baseline e-cigarette use or baseline measurements of the outcome variable were not included. For research question 2, an ordinal logistic mixed regression model was used to determine the association between baseline frequency of e-cigarette use and follow-up frequency of smoking, fit with and without adjustment for covariates, but always adjusting for time, school and baseline smoking frequency.^22^ Finally, for research question 3, on the association between baseline combustible tobacco ever use and ever use of e-cigarettes at follow-ups among never users of e-cigarettes at baseline, a similar analysis protocol as research question 1 was used: a binary logistic mixed regression model predicting e-cigarette use from baseline ever tobacco use status (with and without adjustment for covariates).

To account for missing data, multiple imputation was done with an imputation model that was identical to the analysis model including the covariates. The fully conditional specification method was used,^56^ which has been shown to yield unbiased parameter estimates and SEs.^57 58^ To obtain unbiased estimates of the relations between the predictor variables and the outcomes, missing data on baseline covariates and on the outcome variables were imputed.^59^ An analysis done on imputed data sets introduces power loss and also some uncertainty concerning the p value. By taking the number of imputations at least as large as the proportion of incomplete cases, 80 imputations in the present analysis, this is estimated to yield an acceptable power loss^59^ and a Monte Carlo SE of the p value 0.05 less than 0.01.^55^ Although analysis with multiply imputed data is valid under the assumption of missingness at random,^56^ this assumption cannot be tested. Therefore, for research question 1, a sensitivity analysis was performed using imputations according to a scenario violating this assumption. We chose to impute according to a pessimistic scenario, in that it decreased the association between baseline e-cigarette use and an outcome, by imputing the smoking outcome at follow-up in the same way for both baseline ever and never e-cigarette users. At each follow-up, the highest school-specific incidences of each outcome were taken as smoking probabilities for the imputation. Significance was set to 0.05 and all tests were two tailed. All primary analyses were conducted in Mplus V.7.2.

### Role of funding source

The funder had no role in the study design, the collection or analysis of the data, the interpretation of data, the writing of the report or the decision to submit the article for publication.

## Results

### Study sample

All students in the recruited schools were eligible to participate and enrolled (n=2845). Data were collected for 2839 participants at baseline, including 2185 participants who never smoked tobacco at baseline, of which 1276 (58%) and 1025 (47%) participants completed the 6 and 12-month follow-ups, respectively. The analytical sample available for analyses across waves for research question 1 is depicted in online supplemental figure 1.

### Descriptive analyses

Table 1 shows that boys were more likely than girls to have ever used e-cigarettes. Furthermore, age, highest parental education and all environmental and intrapersonal factors showed significant associations with e-cigarette use at baseline.

Table 2 shows that in the combined sample of ever and never smokers, baseline e-cigarette use was positively associated with baseline use (ever) of combustible tobacco products. Prevalence of combustible tobacco products was between 5.6% (cigars) and 17.8% (cigarettes). 21.6% (n=603) of participants reported ever use of e-cigarettes; 14.7% (n=412) ever use of e-cigarettes as well as combustible tobacco; 7.1% (n=197) combustible tobacco only; and 71.4% (n=1994) never used e-cigarettes nor combustible tobacco.

**Table 2:** Prevalence and cross-sectional association of baseline e-cigarette use and combustible tobacco use in combined sample of baseline ever smokers and never smokers

Baseline ever use	Overall (n=2794)	Baseline e-cigarette never users (n=2191)	Baseline e-cigarette ever users (n=603)	Difference in prevalence rates	
				% (95% CI)	P value
Any combustible tobacco product, n (%)	609 (21.8)	197 (9.0)	412 (68.3)	59.3 (55.4 to 63.2)	<0.001*
Combustible cigarettes, n (%)	496 (17.8)	151 (6.9)	345 (57.2)	50.3 (46.2 to 54.4)	<0.001*
Cigars, n (%)†	157 (5.6)	28 (1.3)	129 (21.4)	20.1 (16.8 to 23.4)	<0.001*
Hookah, n (%)‡	339 (12.2)	81 (3.7)	258 (42.8)	34.1 (30.0 to 38.2)	<0.001*
Number of different combustible tobacco products, n (%)					<0.001*
0	2185 (78.2)	1994 (91.0)	191 (31.7)		
1	324 (11.6)	142 (6.5)	182 (30.2)		
2	187 (6.7)	47 (2.1)	140 (23.2)		
3	98 (3.5)	8 (0.4)	90 (14.9)		

*χ^2^ test.

†Due to missing data for each respective variable, denominators are n=2793.

‡Due to missing data for each respective variable, denominators are n=2790.

### Associations between baseline e-cigarette ever use and combustible tobacco ever use at follow-ups in baseline never smokers

Table 3 shows that, among never smokers, baseline ever e-cigarette users were more likely than never users to have used combustible tobacco at 6-month (23.2% vs 5.5%; % difference=17.7; 95% CI 9.8 to 25.6) and 12-month (44.4% vs 10.8%; % difference=33.6; 95% CI 23.1 to 44.1) follow-ups. Table 4 displays the results after imputation. Interaction of e-cigarette use (ever) with time was significant (OR=3.03; 95% CI 1.39 to 6.63) in the unadjusted analysis. Separate analyses at both follow-ups indicated a stronger association for baseline e-cigarette use and combustible tobacco use at the 12-month follow-up (OR=11.39; 95% CI 6.04 to 21.50), compared with the 6-month follow-up (OR=3.76; 95% CI 2.12 to 6.65). Analysis for time (of follow-up) was significant for both e-cigarette ever and never users, indicating an increase in the use of combustible tobacco across time in both groups. In the adjusted model, the interaction between baseline e-cigarette use and time was also significant for any combustible tobacco product use (OR=2.99; 95% CI 1.37 to 6.50). Furthermore, baseline e-cigarette use was associated more with any combustible tobacco use at 12-month follow-up (OR=5.63; 95% CI 3.04 to 10.42), compared with the 6-month follow-up (OR=1.89; 95% CI 1.05 to 3.37).

**Table 3:** Prevalence of past 6-month combustible tobacco use at 6 and 12-month follow-ups by baseline e-cigarette ever use among baseline never smokers

Use in the prior 6 months		Baseline e-cigarette use		Difference in prevalence rates	
	Overall (n=2185)	Never use (n=1994)	Ever use (n=191)	% (95% CI)	P value
**6-month follow-up**					
Any combustible tobacco product, n (%)*	90 (7.1)	64 (5.5)	26 (23.2)	17.7 (9.8 to 25.6)	<0.001
Combustible cigarettes, n (%)†	63 (4.9)	43 (3.7)	20 (17.9)	14.2 (7.0 to 21.4)	<0.001
Cigars, n (%)‡	15 (1.2)	11 (0.9)	4 (3.6)	2.7 (−0.8 to 6.2)	0.014
Hookah, n (%)§	33 (2.6)	22 (1.9)	11 (9.9)	8.0 (2.4 to 13.6)	<0.001
Number of different combustible tobacco products, n (%)†					<0.001
0	1185 (92.9)	1099 (94.5)	86 (76.8)		
1	72 (5.6)	55 (4.7)	17 (15.2)		
2	15 (1.2)	6 (0.5)	9 (8.0)		
3	3 (0.2)	3 (0.3)	0 (0.0)		
**12-month follow-up**					
Any combustible tobacco product, n (%)¶	141 (13.8)	101 (10.8)	40 (44.4)	33.6 (23.1 to 44.1)	<0.001
Combustible cigarettes, n (%)¶	118 (11.5)	85 (9.1)	33 (36.7)	27.6 (17.5 to 37.7)	<0.001
Cigars, n (%)**	18 (1.8)	14 (1.5)	4 (4.4)	2.9 (−1.4 to 7.2)	0.042
Hookah, n (%)**	47 (4.6)	35 (3.8)	12 (13.3)	9.5 (2.3 to 16.6)	<0.001
Number of different combustible tobacco products, n (%)¶					<0.001
0	884 (86.2)	834 (89.2)	50 (55.6)		
1	106 (10.3)	74 (7.9)	32 (35.6)		
2	28 (2.7)	21 (2.2)	7 (7.8)		
3	7 (0.7)	6 (0.6)	1 (1.1)		

*n=1276.

†n=1275.

‡n=1273.

§n=1270.

¶n=1025.

**n=1023.

**Table 4:** Association of baseline e-cigarette ever use and covariates to combustible tobacco use outcomes at 6 and 12-month follow-ups among baseline never smokers

Baseline regressors and covariates	Outcome									
	Any tobacco product		Combustible cigarettes		Cigars		Hookah		Number of tobacco products	
	OR (95% CI)*	P value	OR (95% CI)*	P value	OR (95% CI)*	P value	OR (95% CI)*	P value	OR (95% CI)†	P value
**Unadjusted models**										
E-cigarette ever (vs never) use										
Smoking outcome at 6 months	3.76 (2.12 to 6.65)	<0.001	4.00 (2.07 to 7.71)	<0.001	3.99 (1.62 to 9.86)	0.003	5.52 (2.74 to 11.12)	<0.001	3.90 (2.22 to 6.85)	<0.001
Smoking outcome at 12 months	11.39 (6.04 to 21.50)	<0.001	9.70 (5.12 to 18.37)	<0.001	3.99 (1.62 to 9.86)	0.003	5.52 (2.74 to 11.12)	<0.001	8.40 (4.64 to 15.21)	<0.001
Time (12 vs 6 months): e-cigarette never	1.98 (1.41 to 2.78)	<0.001	2.43 (1.60 to 3.70)	<0.001	1.53 (0.73 to 3.17)	0.26	2.01 (1.20 to 3.36)	0.008	2.00 (1.43 to 2.82)	<0.001
Time (12 vs 6 months): e-cigarette ever	6.00 (2.98 to 12.11)	<0.001	5.89 (2.79 to 12.44)	<0.001	1.53 (0.73 to 3.17)	0.26	2.01 (1.20 to 3.36)	0.008	4.32 (2.15 to 8.70)	<0.001
Ever e-cigarette use × time‡	3.03 (1.39 to 6.63)	0.006	2.43 (1.07 to 5.48)	0.033	1.44 (0.25 to 8.47)	0.69	1.59 (0.48 to 5.24)	0.45	2.15 (1.01 to 4.58)	0.046
**Adjusted models**										
Categorical covariates										
Girls (vs boys)	0.94 (0.68 to 1.30)	0.73	0.93 (0.65 to 1.33)	0.69	0.65 (0.31 to 1.38)	0.27	0.90 (0.53 to 1.54)	0.71	0.92 (0.67 to 1.27)	0.63
Dutch (vs other) ethnicity	0.42 (0.22 to 0.79)	0.007	0.65 (0.31 to 1.37)	0.26	0.55 (0.16 to 1.88)	0.34	0.22 (0.08 to 0.55)	0.001	0.42 (0.23 to 0.78)	0.006
Lives with both parents (vs other)	0.86 (0.59 to 1.25)	0.42	0.92 (0.63 to 1.36)	0.69	0.75 (0.35 to 1.63)	0.47	0.95 (0.51 to 1.75)	0.86	0.88 (0.62 to 1.26)	0.48
Substance ever (vs never) use	1.73 (1.21 to 2.47)	0.002	1.74 (1.16 to 2.61)	0.007	1.62 (0.74 to 3.56)	0.23	1.54 (0.84 to 2.83)	0.16	1.68 (1.18 to 2.40)	0.004
Family history of smoking (yes vs no)	0.93 (0.67 to 1.29)	0.66	0.93 (0.64 to 1.34)	0.68	0.61 (0.28 to 1.31)	0.20	0.86 (0.49 to 1.50)	0.59	0.93 (0.66 to 1.32)	0.69
Continuous covariates§										
Age	1.13 (0.96 to 1.34)	0.14	1.18 (0.97 to 1.42)	0.10	1.10 (0.75 to 1.63)	0.62	1.08 (0.81 to 1.43)	0.51	1.13 (0.96 to 1.34)	0.15
Parental education	1.00 (0.85 to 1.19)	0.96	1.04 (0.88 to 1.23)	0.65	0.78 (0.54 to 1.12)	0.17	1.10 (0.85 to 1.43)	0.46	1.01 (0.86 to 1.17)	0.95
Peer smoking	1.11 (0.96 to 1.27)	0.17	1.09 (0.94 to 1.25)	0.26	1.03 (0.77 to 1.37)	0.85	1.12 (0.91 to 1.39)	0.28	1.09 (0.96 to 1.25)	0.17
CESD-Depressive symptoms	1.06 (0.91 to 1.24)	0.47	1.09 (0.91 to 1.29)	0.35	1.05 (0.75 to 1.45)	0.79	1.00 (0.80 to 1.25)	1.00	1.07 (0.92 to 1.24)	0.36
TCI-Impulsivity	0.90 (0.77 to 1.06)	0.21	0.90 (0.76 to 1.06)	0.20	0.70 (0.47 to 1.03)	0.07	0.93 (0.73 to 1.17)	0.53	0.89 (0.76 to 1.04)	0.14
Delinquent behaviour	1.03 (0.89 to 1.20)	0.65	1.04 (0.88 to 1.22)	0.66	1.18 (0.93 to 1.50)	0.17	1.15 (0.92 to 1.44)	0.23	1.05 (0.91 to 1.21)	0.53
Smoking susceptibility	1.27 (1.10 to 1.46)	0.001	1.31 (1.13 to 1.52)	<0.001	1.17 (0.85 to 1.62)	0.33	0.91 (0.71 to 1.17)	0.47	1.22 (1.07 to 1.39)	0.002
Smoking expectancies	0.96 (0.81 to 1.13)	0.62	0.90 (0.76 to 1.07)	0.22	1.14 (0.75 to 1.74)	0.54	1.06 (0.81 to 1.39)	0.68	0.97 (0.82 to 1.13)	0.67
Regressors										
E-cigarette ever (vs never) use										
Smoking outcome at 6 months	1.89 (1.05 to 3.37)	0.032	1.93 (0.98 to 3.79)	0.058	1.92 (0.75 to 4.93)	0.18	3.69 (1.75 to 7.77)	0.001	2.04 (1.16 to 3.60)	0.0124
Smoking outcome at 12 months	5.63 (3.04 to 10.42)	<0.001	4.58 (2.42 to 8.68)	<0.001					4.32 (2.40 to 7.76)	<0.001
Time (12 vs 6 months): e-cigarette never	1.98 (1.41 to 2.79)	<0.001	2.44 (1.60 to 3.72)	<0.001	1.53 (0.73 to 3.17)	0.26	2.01 (1.20 to 3.35)	0.008	2.01 (1.43 to 2.83)	<0.001
Time (12 vs 6 months): e-cigarette ever	5.92 (2.95 to 11.87)	<0.001	5.82 (2.77 to 12.19)	<0.001					4.25 (2.12 to 8.55)	<0.001
Ever e-cigarette use × time‡	2.99 (1.37 to 6.50)	0.006	2.38 (1.06 to 5.37)	0.036	1.43 (0.25 to 8.36)	0.69	1.58 (0.48 to 5.20)	0.45	2.12 (1.00 to 4.49)	0.051

All analyses include only never users of combustible tobacco products at baseline (n=2185).

*OR from repeated binary logistic regression model predicting respective outcome from baseline ever e-cigarette use status (yes/no) including school fixed effects.

†OR from repeated ordinal logistic regression model predicting respective outcome from baseline ever e-cigarette use status (yes/no) including school fixed effects, with the OR expressing the change in odds of being in a category with use of a certain amount of tobacco products versus being in a category with lower use (3 vs ≤2, ≥2 vs ≤1, ≥1 vs 0).

‡If interaction term is significant (p≤0.05) or marginally significant (p≤0.10), the effect of e-cigarette use is examined both at 6 and 12- month follow-ups, and the effect of time is examined both for never and ever users of e-cigarettes. If interaction term is not (marginally) significant, the effect of e-cigarette use is examined averaged across and is thus the same for both the 6 and 12-month follow-ups, and the effect of time is examined averaged across and is thus also the same for never and ever users of e-cigarettes.

§Continuous covariates rescaled (M=0, SD=1), such that the ORs indicate change in odds in the outcome associated with an increase in one SD unit on the covariate continuous scale.

CESD, Center for Epidemiologic Studies Depression Scale; TCI, Temperament and Character Inventory.

Table 3 also shows that, among never smokers, e-cigarette use (ever vs never) at baseline was positively associated with higher odds of smoking cigarettes, cigars and hookah. In the unadjusted analyses in table 4, e-cigarette use (ever vs never) at baseline was also positively associated with each of these outcomes at both follow-ups. In the adjusted model, baseline ever e-cigarette use was associated with cigarette use at the 12-month follow-up and with number of tobacco products at both follow-ups. Interaction with time was not significant for hookah use and cigar use, thus associations of baseline ever use of e-cigarettes with these outcomes averaged across time were examined. The relation between e-cigarette use at baseline and hookah use (averaged over 6 and 12-month follow-ups) was significant (OR=3.69; 95% CI 1.75 to 7.77). Of the intrapersonal factors, only smoking susceptibility was positively associated with combustible tobacco use at both follow-ups (see table 4).

Additional sensitivity analyses were performed (see online supplemental table 3). Findings were consistent in the adjusted models for any tobacco use, combustible cigarette use, cigar use and number of tobacco products. For hookah use, there was a significant interaction of e-cigarette use and time. Only the association between e-cigarette use and hookah use at 6-month follow-up remained significant.

### Association between baseline e-cigarette use frequency and tobacco use frequency at follow-ups

Online supplemental table 1 shows that, for the unadjusted model, higher scores on the four-level baseline e-cigarette use frequency variable were associated with greater odds of higher smoking frequency averaged across both follow-ups (OR=2.11; 95% CI 1.69 to 2.64). After adjusting for covariates, this association remained significant (OR=1.63; 95% CI 1.29 to 2.06). In the unadjusted analysis, the positive association between baseline e-cigarette use and follow-up smoking frequency differed between the different baseline smoking groups (OR=0.70; 95% CI 0.52 to 0.94, p=0.02), the association being stronger among baseline non-smokers (n=2180; OR=2.53; 95% CI 1.99 to 3.23) than baseline infrequent (smoked 1–2 in the past 30 days; n=41; OR=1.84; 95% CI 1.46 to 2.31) and frequent (smoked ≥3 in the past 30 days; n=127; OR=1.33; 95% CI 0.89 to 1.99) smokers (not shown in online supplemental table 1).

### Association between baseline ever smoking and follow-up ever e-cigarette use at follow-ups in baseline never e-cigarette users

Online supplemental table 2 shows that among never users of e-cigarettes, smoking at baseline was positively associated with higher odds of using e-cigarettes averaged across both follow-ups for the unadjusted (OR=5.22; 95% CI 3.06 to 8.92) and adjusted (OR=3.10; 95% CI 1.58 to 6.06) analyses.

## Discussion

The current replication study confirms that e-cigarette use by non-smoking adolescents is associated with increased odds of subsequent combustible tobacco smoking initiation; that more frequent e-cigarette use is associated with more frequent subsequent tobacco smoking; and that the ‘reverse’ association applies, namely that tobacco smoking among never users of e-cigarettes is associated with greater odds of later e-cigarette use. These findings are consistent with previous studies,^21 22 30 31^ employ various adjusted analyses and sensitivity analyses and extend the findings from the US to a European context.

Collectively, this suggests that e-cigarettes may indeed act as a gateway to tobacco smoking for youth, as we see that youth who used e-cigarettes are more likely to try smoking tobacco later. We also found a dose-related relation, namely that the higher the frequency of e-cigarette use was at baseline, the higher the frequency of subsequent tobacco use was at follow-up. This may indicate a causal relation for using both products. Additionally, our analyses suggest a ‘reversed’ gateway of tobacco smoking to e-cigarette use. Yet, these gateways may operate through different mechanisms. E-cigarettes may be a smoother introduction to tobacco smoking^60^; and reversely, the transition from tobacco smoking to e-cigarette use could point at transitioning to a less harmful behaviour. However, both behaviours may also stem from common risk factors, such as increased propensity to experiment with substances,^14 61–63^ where final choices on which specific substance is used first may depend on personal preferences, circumstances or cultural norms.^32^ Dual users of e-cigarettes and cigarettes, for example, share similar characteristics (eg, impulsivity) regardless of which product is used first.^64^

It remains uncertain whether the found associations are causal, which the gateway hypothesis suggests, or an indication of shared risk factors for e-cigarette and tobacco use. While multiple predictors linked to tobacco smoking were addressed in our study, it remains uncertain whether all common liability risks were controlled for. Studies show that at least some youth with low propensity for tobacco smoking, nevertheless, used e-cigarettes.^24 65 66^ Another study found that relative to a propensity-matched control group without initial e-cigarette use, non-smoking adolescent e-cigarette users were less likely to become established smokers (30-day use and 100+ lifetime cigarettes).^67^ This suggests that e-cigarettes do not function as a gateway to tobacco for everyone. Additionally, a study found that the relationship between e-cigarette use and subsequent smoking among adolescents may be weakened through interventions.^68^ Likely, multiple mechanisms are complementary and the relation between causes and outcomes is complex and multidirectional.^69^

A critique on quantitative studies on the gateway from e-cigarette to tobacco smoking is that the key question of ‘why’ is not addressed.^63^ A popular explanation is that the nicotine in e-cigarettes makes individuals dependent and this may cause them to try combustible cigarettes.^70 71^ The authors of the original gateway theory even describe nicotine as a gateway drug that primes the brain for other substance use, ‘whether the exposure is from smoking tobacco, passive tobacco smoke, or e-cigarettes.’^72^ A qualitative study of youth who use(d) e-cigarettes found that e-cigarettes can be a ‘smoother’ introduction to the concept of smoking and that they appear to remove boundaries to smoking: ‘There used to be a barrier that said either you’re a smoker or a non-smoker, now I can smoke without smoking’.^60^ Consequently, e-cigarette users get used to the acts and gestures of smoking which facilitates the transition to tobacco smoking. However, information in this area is still limited and more investigation is needed, including studies among tobacco smokers who started with e-cigarettes to assess reasons for taking up smoking.

Evidence and understanding of associations between e-cigarettes and tobacco and the long-term consequences of e-cigarette use among youth are still limited,^28^ partly because of the relative novelty of e-cigarettes and still evolving technologies. Until this has been resolved, policymakers should carefully consider whether to act on the dangers (eg, a gateway to tobacco) or rather the benefits (eg, a potentially less harmful alternative to tobacco smoking) of e-cigarettes for smokers. Given the wide availability and marketing of e-cigarettes^73 74^ and that available evidence provides reasons for caution, prevention of e-cigarette use among non-smoking youth is recommended.

### Limitations

The original and current study used binary and categorical outcome measures of smoking. These measures are limited, as smoking intensity (how many cigarettes per day) was not assessed and a fairly low cut-off point for frequent smoking (≥3 days during the past 30 days) was used because of the limited number of participants. Also, the majority (68.3%) of ever users of e-cigarettes had already smoked tobacco at baseline, which may mean that the current study sample is already too old for the main research question focusing on never users of tobacco. Furthermore, substance use was included as a dichotomous variable, meaning, for example, that students who drank alcohol and used cannabis on a weekly basis got the same score as students who had one drink in their lifetime. Additionally, the response of participating (vs invited) schools was low, which may have resulted in selection bias. However, relevant differences between students from participating schools and students at non-participating schools are not expected, as the response rate of students who were present at the time of the survey in participating school classes was 100%. Furthermore, the dropout rate from baseline to follow-up was high and may have affected the relations between e-cigarette use and tobacco use. However, the imputation of missing outcomes for research question 1 according to a pessimistic scenario (see the Data analysis section) yielded the same relations, indicating robustness of the results. Lastly, we coded Flemish (Belgian) students as Dutch in the main analyses because they are part of the (native) ethnic majority of their schools. Thus, differences between Dutch and Belgian students were not assessed, even though (cultural) differences may exist. However, preliminary analyses, in which Flemish students were coded as ‘not Dutch’, yielded similar results in the main analyses.

## Conclusion

For this study, we replicated an American cornerstone study^21 22^ on the association between e-cigarette use and tobacco smoking in the Netherlands and Flanders and found similar results. High school students in the Netherlands and Flanders who used e-cigarettes at baseline were more likely to report initiation of combustible tobacco use over the next year compared with never users of e-cigarettes. These findings add to a growing body of studies that indicate a link between e-cigarette use and tobacco smoking in youth. The gateway hypothesis was further explored by also analysing the ‘reverse’ relation between baseline tobacco smoking and subsequent e-cigarette use. A similar association was found which may indicate that the gateway works in two directions, that e-cigarette use and tobacco smoking share common risk factors, or that both mechanisms apply. Different types of studies are needed to better understand *why* there are such associations and whether these may be causal relationships.

**What is already known on this subject**

- E-cigarette use is associated with subsequent tobacco smoking among youth.
- A popular explanation of this relation is that e-cigarettes function as a gateway to tobacco smoking for youth.

**What important gaps in knowledge exist on this topic**

- Most studies only investigate the relation between e-cigarette use and tobacco smoking in one direction. Less is known about the opposite relation between tobacco smoking and subsequent e-cigarette use.
- It is not yet determined whether the gateway hypothesis or the common liability hypothesis best explains the relation between e-cigarette use and tobacco smoking.

**What this paper adds**

- This study replicated the findings from an American cornerstone study on the relationship between e-cigarette use and tobacco use. Findings indicate both the relationship between e-cigarette use and subsequent tobacco smoking and the opposite relation.
- The association between e-cigarette use and tobacco smoking is likely bidirectional for adolescents.

## Data Availability

Data are available upon reasonable request. Anonymised data and protocols are available upon reasonable request from the corresponding author (martinelli@ivo.nl).
