# Supplementary material for: Exploring the gateway hypothesis of e-cigarettes and tobacco: a prospective replication study among adolescents in the Netherlands and Flanders
Source: Tob Control. 2021 Jul 5;32(2):170–8. doi: 10.1136/tobaccocontrol-2021-056528 (PMC9985733; doi:10.1136/tobaccocontrol-2021-056528)
Supplement: Supplementary data [file tobaccocontrol-2021-056528supp001.pdf]

Supplementary tables and figures:

Supplementary Figure 1: Flow of Adolescent Students in Study to Assess E-Cigarette Use at Baseline and Later Use of Combustible Tobacco Products

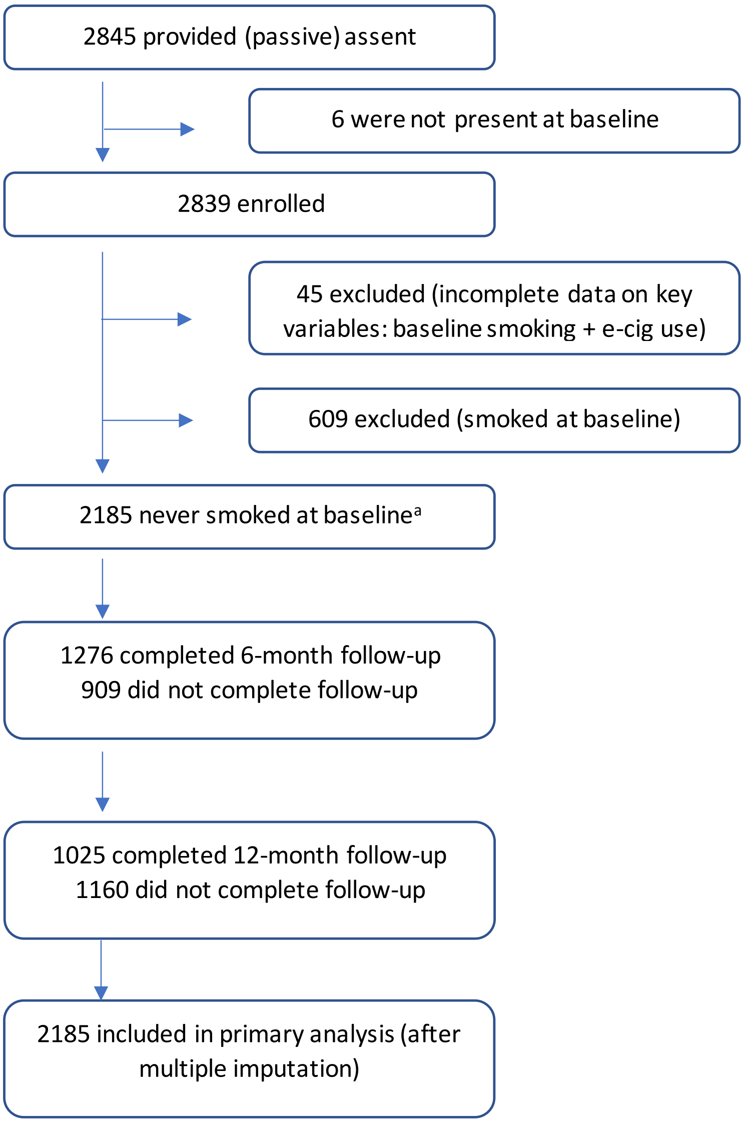

[a] Includes all combustible tobacco products.

Supplementary Table 1: Association of Baseline e-cigarette use frequency with tobacco smoking frequency at 6- and 12-month Follow-up[a]

	Parameter Estimate for Association with Smoking Frequency at follow-up	
	Odds ratio (95% CI)	P-value
**Unadjusted model**[b]		
Baseline smoking (3-level continuum):		
- Outcome at 6 months	2.06 (1.41, 3.01)	< 0.001
- Outcome at 12 months	7.11 (4.03, 12.56)	< 0.001
E-cigarette use (4-level continuum)	2.11 (1.69, 2.64)	< 0.001
E-cigarette use x smoking[c]	0.70 (0.52, 0.94)	0.02
**Adjusted model**[d]		
Baseline smoking (3-level continuum)		
- Outcome at 6 months	1.11 (0.71, 1.73)	0.66
- Outcome at 12 months	3.52 (2.02, 6.11)	<0.001
E-cigarette use (4-level continuum)	1.63 (1.29, 2.06)	< 0.001
E-cigarette use x smoking[c]	0.83 (0.61, 1.11)	0.21

[a] Ordinal logistic mixed regression of proportional odds for being at a higher smoking frequency outcome (i.e., days smoked in the past 30 days; non-smoker, 0; infrequent smokers [1-2days], 1; frequent smokers [≥3 days], 2) accounting for clustering by pupil and school in a sample with available baseline e-cigarette use and smoking frequency data (N = 2348). The baseline 4-level continuous e-cigarette use variable was categorized as never, prior (ever with no past 30-day use), infrequent (1-2 days during past 30 days), or frequent (≥3 days during past 30 days). To address missing covariate data and missing outcomes, 80 multiple imputed datasets were generated using the fully conditional specification method (with sequential regression procedure). The parameter estimates from models in each imputed data set were pooled and presented as a single estimate.

[b] Unadjusted models without e-cigarette use x smoking interaction include baseline e-cigarette use and smoking frequency, and interactions of these variables with time (6 versus 12 months follow-up). In case of a significant interaction with time, parameter estimates are shown separately for 6- and 12-months follow-up, otherwise the interaction term is removed from the model.

[c] Interaction term added in subsequent model. If e-cigarette use x smoking x time is not significant, the results for e-cigarette use x smoking averaged across both follow-ups are presented.

[d] Adjusted models without e-cigarette use x smoking interaction include baseline e-cigarette use and smoking frequency, and interactions of these variables with time (6 versus 12 months follow-up). In case of a significant interaction with time, parameter estimates are shown separately for 6- and 12-months follow-up, otherwise the interaction term is removed from the model. Adjustment is made for demographic, environmental and psychosocial covariates as described in the main text.

Supplementary Table 2: Association of Baseline Ever Use of Any Tobacco Product and Covariates to E-cigarette Use at 6- and 12-Month Follow Ups among Baseline Never E-Cigarette Users

Baseline regressors and covariates	Ever Use of E-Cigarettes	
	OR(95%*CI*) [a]	*P*
**Unadjusted Models**		
Any Tobacco Product ever (vs. never) use:		
E-Cigarette use at 6 months	5.22 (3.06,8.92)	< 0.001
E-Cigarette use at 12 months	5.22 (3.06,8.92)	< 0.001
Time (12- vs. 6-month): Any Tobacco use never	1.46 (1.01,2.12)	0.04
Time (12- vs. 6-month): Any Tobacco use ever	1.46 (1.01,2.12)	0.04
Ever Tobacco Product Use × Time [b]	1.92 (0.79,4.65)	0.15
**Adjusted Models**		
Categorical covariates		
Girls (vs. Boys)	0.83 (0.56,1.22)	0.33
Dutch (vs. other) ethnicity	0.82 (0.33,2.06)	0.67
Lives with both parents (vs. other)	0.90 (0.59,1.39)	0.64
Substance ever (vs. never) use	2.03 (1.30,3.16)	0.002
Family history of smoking (yes vs. no)	0.97 (0.65,1.45)	0.88
Continuous covariates [c]		
Age	1.02 (0.83,1.25)	0.84
Parental education	0.99 (0.83,1.18)	0.95
Peer smoking	0.98 (0.83,1.17)	0.86
CESD-Depressive Symptoms	0.92 (0.76,1.12)	0.47
TCI-Impulsivity	0.97 (0.81,1.16)	0.71
Delinquent Behaviour	1.11 (0.96,1.29)	0.17
Smoking susceptibility	1.04 (0.85,1.26)	0.71
Smoking expectancies	0.98 (0.79,1.20)	0.82
Regressors		
Any Tobacco Product ever (vs. never) use:		
E-Cigarette use at 6 months	3.10 (1.58,6.06)	0.001
E-Cigarette use at 12 months	3.10 (1.58,6.06)	0.001
Time (12- vs. 6-month): Any Tobacco use never	1.47 (1.01,2.12)	0.04
Time (12- vs. 6-month): Any Tobacco use ever	1.47 (1.01,2.12)	0.04
Ever Tobacco Product Use × Time [b]	1.91 (0.79,4.63)	0.15

Note: All analyses include only never-users of E-Cigarettes at baseline (*N* = 2191).
[a] *OR* from repeated binary logistic regression model predicting E-cigarette use from baseline ever tobacco use status (yes/no) including school fixed effects.
[b] If interaction term is significant (p-value ≤ 0.05), the effect of any tobacco product use is examined both at 6- and 12 months follow up, and the effect of time is examined both for never and ever users of any tobacco product. If interaction term is not significant, the effect of any tobacco use is examined averaged across and, is thus the same for both the 6- and 12 months follow ups, and the effect of time is examined averaged across and, is thus also the same for never and ever users of tobacco products.
[c] Continuous covariates rescaled (*M*= 0, *SD*=1), such that the *OR*s indicate change in odds in the outcome associated with an increase in one standard deviation unit on the covariate continuous scale. CESD = Center for Epidemiologic Studies Depression Scale. TCI = Temperament and Character Inventory.

Supplementary Table 3: Sensitivity analyses of the Association of Baseline E-Cigarette Ever Use and Covariates to Combustible Tobacco Use Outcomes at 6- and 12-Month Follow Ups among Baseline Never-Smokers

Baseline regressors and covariates	Outcome									
	Any tobacco product		Combustible cigarettes		Cigars		Hookah		Number of Tobacco Products	
	OR (95%*CI*) [a]	*P*	*OR* (95%*CI*) [a]	*P*	*OR* (95%*CI*) [a]	*P*	*OR* (95%*CI*) [a]	*P*	*OR* (95%*CI*) [b]	*P*
Unadjusted Models										
E-cigarette ever (vs. never) use:										
Smoking outcome on 6 months	2.33 (1.75,3.11)	<0.001	2.62 (1.85,3.71)	<0.001	1.43 (0.90,2.25)	0.13	3.00 (1.61,5.60)	0.001	2.30 (1.73, 3.08)	<0.001
Smoking outcome on 12 months	2.33 (1.75,3.11)	<0.001	2.62 (1.85,3.71)	<0.001	1.43 (0.90,2.25)	0.13	1.28 (0.71,2.32)	0.41	2.30 (1.73,3.08)	<0.001
Time (12- vs. 6-month): E-Cigarette never	2.77 (2.33,3.30)	<0.001	3.16 (2.55,3.91)	<0.001	2.32 (1.69,3.19)	< 0.001	2.34 (1.73,3.18)	< 0.001	2.80 (2.35,3.33)	<0.001
Time (12- vs. 6-month): E-Cigarette ever	2.77 (2.33,3.30)	<0.001	3.16 (2.55,3.91)	<0.001	2.32 (1.69,3.19)	< 0.001	1.00 (0.49,2.06)	1.00	2.80 (2.35,3.33)	<0.001
Ever e-cigarette use × Time [c]	0.95 (0.57,1.57)	0.85	0.98 (0.56,1.73)	0.96	0.71 (0.26,1.92)	0.50	0.43 (0.20,0.93)	0.033	0.85 (0.52,1.39)	0.51
Adjusted Models										
Categorical covariates										
Girls (vs. Boys)	0.97 (0.81,1.16)	0.75	0.91 (0.73,1.15)	0.44	0.91 (0.66,1.25)	0.56	1.02 (0.76,1.38)	0.89	0.95 (0.80,1.14)	0.60
Dutch (vs. other) ethnicity	0.59 (0.38,0.91)	0.02	0.68 (0.41,1.12)	0.13	1.07 (0.48,2.42)	0.87	0.33 (0.17,0.65)	0.001	0.57 (0.36,0.90)	0.02
Lives with both parents (vs. other)	1.10 (0.88,1.37)	0.40	1.27 (0.96,1.67)	0.10	0.93 (0.65,1.34)	0.71	1.11 (0.76,1.61)	0.59	1.12 (0.90,1.40)	0.31
Substance ever (vs. never) use	1.28 (1.04,1.56)	0.02	1.21 (0.95,1.56)	0.13	1.47 (1.05,2.05)	0.03	1.39 (0.99,1.94)	0.06	1.30 (1.07,1.59)	0.01
Family history of smoking (yes vs. no)	1.12 (0.92,1.37)	0.24	1.05 (0.83,1.34)	0.68	0.98 (0.70,1.38)	0.93	1.11 (0.80,1.54)	0.55	1.10 (0.90,1.33)	0.35
Continuous covariates [d]										
Age	1.32 (1.19,1.46)	< 0.001	1.25 (1.10,1.41)	< 0.001	1.26 (1.06,1.51)	0.01	1.24 (1.05,1.46)	0.01	1.30 (1.18,1.44)	< 0.001
Parental education	0.98 (0.89,1.09)	0.76	1.04 (0.92,1.17)	0.55	0.90 (0.75,1.07)	0.23	1.03 (0.87,1.21)	0.73	0.99 (0.90,1.09)	0.85
Peer smoking	1.09 (1.00,1.19)	0.04	1.09 (0.98,1.20)	0.11	1.03 (0.91,1.17)	0.67	1.07 (0.94,1.22	0.29	1.09 (1.00,1.18)	0.06
CESD-Depressive Symptoms	1.07 (0.97,1.17)	0.18	1.09 (0.97,1.22)	0.16	1.06 (0.90,1.25)	0.48	1.01 (0.88,1.16)	0.88	1.07 (0.98,1.17)	0.14
TCI-Impulsivity	0.84 (0.77,0.92)	< 0.001	0.79 (0.71,0.88)	< 0.001	0.82 (0.70,0.96)	0.01	0.93 (0.80,1.07)	0.32	0.83 (0.76,0.91)	< 0.001
Delinquent Behaviour	1.05 (0.95,1.16)	0.35	1.09 (0.97,1.24)	0.15	1.00 (0.86,1.17)	0.98	1.08 (0.93,1.25)	0.32	1.06 (0.96,1.18)	0.24
Smoking susceptibility	1.12 (1.02,1.23)	0.02	1.17 (1.04,1.30)	0.01	0.98 (0.82,1.17)	0.80	0.95 (0.82,1.10)	0.46	1.10 (1.01,1.20)	0.04
Smoking expectancies	0.94 (0.86,1.03)	0.16	0.92 (0.82,1.02)	0.12	1.01 (0.85,1.20	0.90	1.01 (0.86,1.18)	0.91	0.95 (0.87,1.04)	0.26
Regressors										
E-cigarettes ever (vs. never) use:										
Smoking outcome on 6 months	1.37 (1.01,1.84)	0.042	1.49 (1.05,2.12)	0.028	0.98 (0.59,1.61)	0.93	0.47 (0.25, 0.89)	0.02	1.36 (1.01,1.83)	0.045
Smoking outcome on 12 months	1.37 (1.01,1.84)	0.042	1.49 (1.05,2.12)	0.028	0.98 (0.59,1.61)	0.93	0.91 (0.50, 1.68)	0.77	1.36 (1.01,1.83)	0.045
Time (12- vs. 6-month): E-cigarette never	2.78 (2.34,3.31)	<0.001	3.16 (2.56,3.92)	<0.001	2.29 (1.67,3.15)	<0.001	2.35 (1.73,3.19)	< 0.001	2.80 (2.35,3.32)	<0.001
Time (12- vs. 6-month): E-cigarette ever	2.78 (2.34,3.31))	<0.001	3.16 (2.56,3.92)	<0.001	2.29 (1.67,3.15)	<0.001	1.00 (0.48,2.10)	1.00	2.80 (2.35,3.32)	<0.001
Ever e-cigarette use × Time [c]	0.95 (0.57,1.58)	0.85	0.99 (0.56,1.74)	0.96	0.71 (0.26,1.93)	0.50	0.43 (0.19,0.93)	0.033	0.84 (0.51,1.37)	0.48

Note: Sensitivity analysis: for missing outcomes values were imputed assuming the prevalence for each of these outcomes at 6 and 12-month follow-up as found in schools with the highest prevalence on each of these outcomes. All analyses include only never-users of combustible tobacco products at baseline (*N* = 2185).

[a] *OR* from repeated binary logistic regression model predicting respective outcome from baseline ever e-cigarette use status (yes/no) including school fixed effects.

[b] *OR* from repeated ordinal logistic regression model predicting respective outcome from baseline ever e-cigarette use status (yes/no) including school fixed effects, with the OR expressing the change in odds of being in a category with use of a certain amount of tobacco products versus being in a c ategory with lower use (3 versus ≤ 2, ≥ 2 versus ≤ 1, ≥ 1 versus 0).

[c] If interaction term is significant (p-value ≤ 0.05) or marginally significant (p-value ≤ 0.10), the effect of e-cigarette use is examined both at 6- and 12 months follow up, and the effect of time is examined both for never and ever users of e-cigarettes. If interaction term is not (marginally) significant, the effect of e-cigarette use is examined averaged across and, is thus the same for both the 6- and 12 months follow ups, and the effect of time is examined averaged across and, is thus also the same for n ever and ever users of e-cigarettes.

[d] Continuous covariates rescaled (*M*= 0, *SD*=1), such that the *OR*s indicate change in odds in the outcome associated with an increase in one standard deviation unit on the covariate continuous scale. CESD = Center for Epidemiologic Studies Depression Scale. TCI = Temperament and Character Inventory.
